# Supplementary material for: Exploring Renal Changes after Bariatric Surgery in Patients with Severe Obesity
Source: J Clin Med. 2022 Jan 29;11(3):728. doi: 10.3390/jcm11030728 (PMC8837063; doi:10.3390/jcm11030728)
Supplement: Supplementary file 1 [file jcm-11-00728-s001.zip › jcm-1555308-supplementary.pdf]

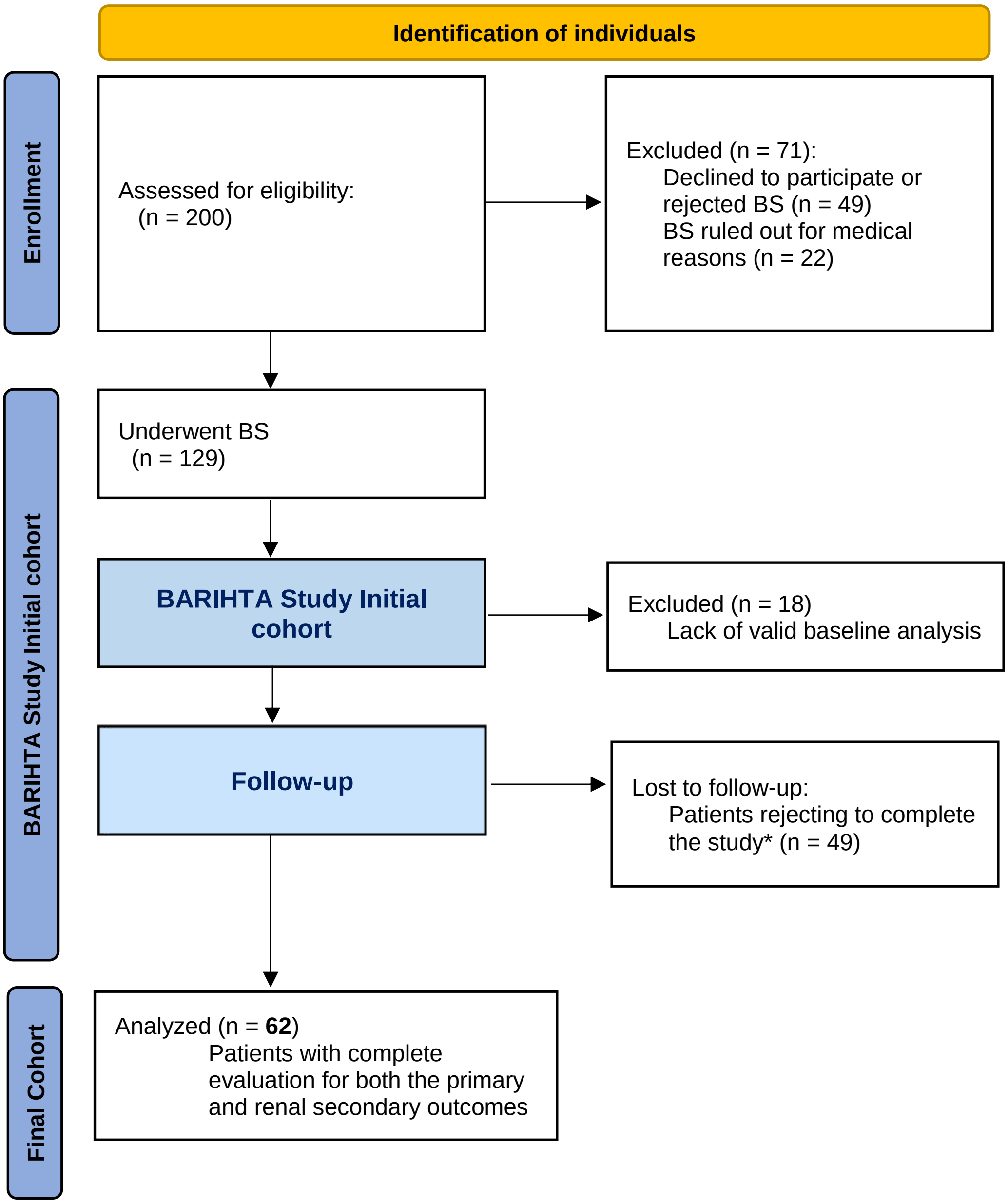

BS = Bariatric surgery

Figure S1. Flowchart for participants in the BARIHTA Study. * Most patients rejected to complete the study because of complains about wearing the 24h-Mobile-O-Graph® device or due to work reasons (having achieved work reinsertion after losing weight)

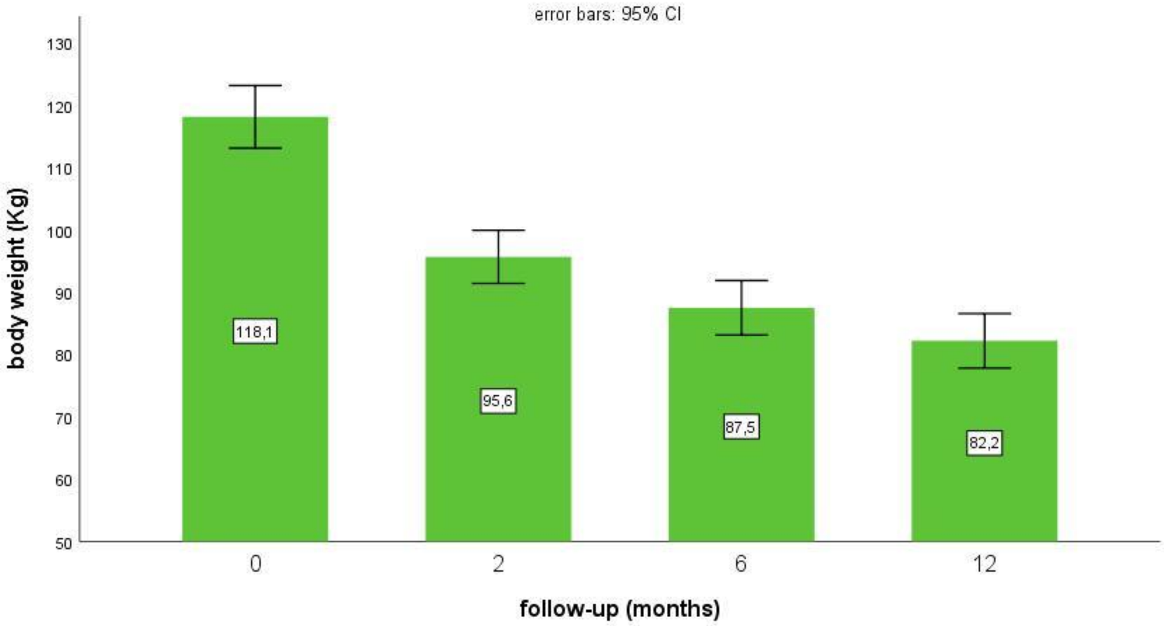

Figure S2. Body weight change at follow-up.

Table S1. Changes in adipokines and inflammatory markers 12-months after bariatric surgery.

	Before BS	12- months post-BS	p
**Leptin*,** ng/mL	56.5 [28.8; 73.7]	12.8 [8.1; 33.2]	**<0.001**
**Adiponectine*,** μg/mL	19.0 [12.5; 33.3]	29.0 [15.5; 59.0]	**0.017**
**Resistin,** ng/mL	37.4 ± 15.9	33.6 ± 12.1	0.091
**MCP-1,** pg/mL	556.6 ± 204.1	554.0 ± 219.8	0.930
**Angiopoietin2*,** μg/mL	2676.2 [1815.4; 4067.0]	4540.5 [2094.2; 6190.4]	**0.012**
**hs-CRP*,** mg/dL	0.77 [0.43; 1.41]	0.11 [0.06; 0.29]	**<0.001**

(*) Data shown as median [interquartile range]

BS = bariatric surgery; **hs-CRP** = C-reactive protein; **MCP-1** = monocyte chemoattractant protein-1

Table S2. Determinants of variation of eGFR (S2A) and of variation of albuminuria (S2B)

S2A.

eGFR	All patients (n = 62)			Patients without antihypertensive treatment (n = 42)		
	**Coeff.**	**95% CI**	***p*-value**	**Coeff.**	**95% CI**	***p*-value**
**Months FU**	-0.56	-1.98, 0.87	0.443	-0.95	-2.88, 0.99	0.337
**Body weight, Kg**	0.66	0.35, 0.96	**<0.001**	0.50	0.11, 0.88	**0.012**
**24h-systolic BP, mmHg**	0.32	-0.04, 0.67	0.082	-0.04	-0.54, 0.46	0.872
**Aldosterone, ng/dL**	-0.10	-0.15, -0.05	**<0.001**	-0.06	-0.12, -0.00	**0.033**
**HOMA** index	0.47	-0.62, 1.57	0.398	1.25	0.13, 2.37	**0.029**

BP = blood pressure; **eGFR** = estimated glomerular filtration rate; **FU** = follow-up; **HOMA** = homeostasis model assessment-estimated insulin resistance.

S2B.

lnACR	All patients (n = 62)			Patients without antihypertensive treatment (n = 42)		
	**Coeff.**	**95% CI**	***p*-value**	**Coeff.**	**95% CI**	***p*-value**
**Months FU**	-0.02	-0.07, 0.02	0.278	-0.04	-0.10, 0.01	0.119
**Body weight, Kg**	-0.00	-0.01, 0.00	0.364	-0.00	-0.01, 0.01	0.516
**24h-systolic BP, mmHg**	0.02	0.01, 0.03	**<0.001**	-0.01	-0.02, 0.01	0.333
**Aldosterone, ng/dL**	0.00	--0.00, 0.00	0.430	0.00	-0.00, 0.00	0.980
**HOMA** index	0.05	0.01, 0.08	**0.012**	0.04	0.01, 0.07	**0.022**

BP = blood pressure; **FU** = follow-up; **HOMA** = homeostasis model assessment-estimated insulin resistance; **lnACR** = log-transformed albumin-creatinine ratio.
